# A new approach based on isoindole formation reaction for sensitive fluorimetric assay of milnacipran in tablets and biological fluids (plasma/urine)

**DOI:** 10.1039/d0ra05162d

**Published:** 2020-10-23

**Authors:** Ahmed A. Abu-hassan, Ramadan Ali, Sayed M. Derayea

**Affiliations:** Department of Pharmaceutical Analytical Chemistry, Faculty of Pharmacy, Al-Azhar University, Assiut Branch Assiut 71524 Egypt ahmedabuhassan@azhar.edu.eg; Department of Analytical Chemistry, Faculty of Pharmacy, Minia University Minia 61519 Egypt

## Abstract

The current study describes a new, sensitive, and economic protocol for milnacipran analysis. Milnacipran was introduced as a therapy for fibromyalgia and depression. It acts by unique and equal inhibition of noradrenaline and serotonin neurotransmitters reuptake. In the presence of 2-mercaptoethanol, the amino moiety of milnacipran condenses with *o*-phthalaldehyde to generate isoindole fluorescent derivative. The isoindole product was measured at (*λ*_ex_ 338.5 nm, *λ*_em_ 433.5 nm) and condensation variables were strictly optimized. The fluorescence intensity of measurements was plotted *versus* milnacipran concentration to give a linearity range over 200–4000 ng mL^−1^. The proposed approach was fully validated by the directives of ICH guidelines and applied without any influence of combined excipient for milnacipran tablet analysis. Furthermore, the procedure was applied in spiked urine and plasma analysis with excellent percentage recovery.

## Abstract 2

The current study describes a new, sensitive, and economic protocol for milnacipran analysis.

## Graphical abstract

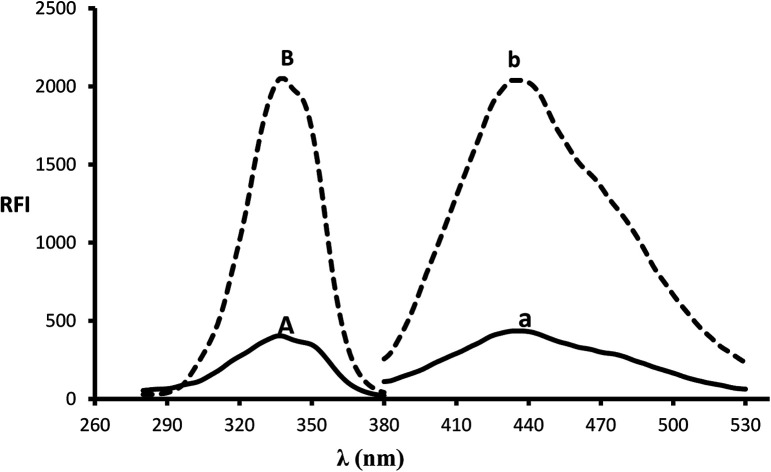

## Introduction

Among medical problems that affect many people are fibromyalgia and depression. Fibromyalgia is a complicated disease characterized by many symptoms as generalized chronic muscular pain, sleep disturbance, anxiety, fatigue, cognitive dysfunction, and stiffness.^1^ About 2–4% of people are affected by fibromyalgia.^2^ Depression is a mood condition that is substantially different from normal mood changes in daily life. Many symptoms are accompanied by depression as weight loss, psychomotor retardation, insomnia, concentration problems, fatigue, and anxiety. In severe states, patients may have a feeling of worthlessness, hallucinations, and thoughts of suicide.^3^ Milnacipran (MLC; 2-(aminomethyl)-*N*,*N*-diethyl-1-phenylcyclopropane-1-carboxamide) was accepted by the FDA as a therapy for depression and fibromyalgia.^4^ It acts by unique and equal inhibition of noradrenaline and serotonin neurotransmitters reuptake.^5^

In the previous analytical literature, MLC was assayed by various techniques which include GC-MS,^6^ liquid chromatography with fluorescence or UV detection,^7,8^ spectrofluorimetry by derivatization with ninhydrin^9^ or NBD-Cl,^10^ UV-spectrophotometric detection at 223 nm^11,12^ and colorimetry using ninhydrin^13^ or ion pair complex.^14^

The approach target was simple development for accurate assay of MLC in the spiked biological fluid, and tablets. The method provides advantages over the published method as the heating step is absent which make the method is more facile than the reported methods,^9,10^ non-extractive,^13,14^ low LOD and LOQ,^9,11,13,14^ and finally implemented in non-sophisticated and easily available equipment.

The assay strategy simply relies on isoindole formation reaction. At suitable basic pH, isoindole reaction is the fundamental concept of many derivative reactions used in spectrofluorimetric research.^15–18^ The approach is considered an alternative way for MLC analysis with many advantages. The directives that followed for application and validation were ICH guidelines.

## Experimental

### Apparatus

Throughout the work, fluorescence intensities were monitored by a spectrofluorimeter FS-2 (Scinco, Korea). The instrument is equipped with Xe-arc lamp (150 W). The voltage of PMT and both monochromators (excitation and emission) slit width and were set at 400 V and 5 nm respectively.

### Chemicals and materials

We have been supplied with Milnacipran hydrochloride from Mash Premiere (Badr City, Egypt) pharmaceutical company as a gift. Milnavella (Mash Premiere) and Averomilan® (Averroes-pharma, Egypt) with 50 mg active milnacipran were utilized in dosage form analysis. *o*-Phthalaldehyde (Alpha Chemika, India) was prepared freshly by dissolving 10 mg in 5 mL methanol and volume adjusted by water to 100 mL to give concentration 7.46 × 10^−4^ M. 2-Mercaptoethanol (99%) was purchased from Sigma (MO, USA), stored at 2–8 °C and diluted by water to give (0.01% v/v) solution. Organic solvent, boric acid, and sodium hydroxide were obtained from El-Nasr chemical-Co (Cairo, Egypt). Borate buffer (0.2 M) solution was prepared by combining suitable volumes of 0.2 M NaOH and 0.2 M boric acid and adjusting the intended pH by pH meter. Human plasma and urine samples were obtained from healthy male volunteers with ages ranged from 30–39 years old at Assiut University Clinics according to institutional guidelines. In all cases, informed written consent was obtained from all participants and plasma samples were kept frozen at −20 °C until assessment after gentle thawing. Urine samples were stored in the refrigerator at 4 °C until analysis.

### MLC standard solution

Stock MLC solution was prepared by dissolving 20 mg of MLC powder in a 100 mL flask. The powder was initially dissolved in 25 mL distilled water then completed to the mark with the same solvent. Different MLC working solutions were prepared by further dilution of the previously prepared stock solution.

### General analytical procedure

The procedure was implemented in 10 mL flask. To each flask 1.4 mL borate buffer (pH = 10.5) was mixed with an aliquot volume of MLC equal to (200–4000 ng) in the final working solution. The content was thoroughly mixed with 1.2 mL of 2-mercaptoethanol (0.01 v/v) followed by 1 mL *o*-phthalaldehyde (7.46 × 10^−4^ M), then allowed to stand 25 minutes. Finally, the final volume was adjusted with methanol and the fluorescence intensity of the isoindole product was recorded at (*λ*_ex_ 338.5 nm, *λ*_em_ 433.5 nm). All fluorescence reading was corrected against an analytical blank prepared simultaneously.

### Tablet formulation analysis

Ten tablets of Milnavella or Averomilan® were weighed and crushed entirely to a fine powder. A quantity of dosage form powder equal to 50 mg MLC was placed and sonicated in the flask with 30 mL distilled water for 15 min. The content of the flask was accurately filtered into another volumetric flask and completed to the mark by water. After suitable dilution, the percentage recovery of tablets content was estimated from the regression equation after analysis by the recommended approach procedure.

### Steps for spiked human plasma

At Minia University Hospital, the process of plasma isolation was implemented. The blood was taken from healthy volunteers (who not taken MLC) *via* the forearm vein, then placed in heparinized tubes. Plasma isolation was accomplished by centrifugation of the tube content at 5000 rpm for 20 min. Until the time of the assay, the plasma was held at −20 °C.^19^ Into a series of Eppendorf tubes (5 mL) 800 μL of the stored plasma and 200 μL of MLC (containing the suitable concentration) was mixed by the vortex. Separation of plasma protein was performed by adding 3 mL of acetonitrile followed by centrifugation at the time and value mentioned above. Ultimately, 1.0 mL from the tube content was transferred into a 10 mL analytical flask and was analyzed by the recommended procedure and the response was corrected against plasma blank.

### MLC assay in spiked urine

Into 50 mL separating funnel, 800 μL human urine, 200 μL MLC solution (containing the different concentrations), and 3 mL NaOH/carbonate solution (pH = 11.8) was added. Then the solution was extracted three times using 5 mL dichloromethane in each one. The collected organic layer was carefully evaporated under nitrogen. The MLC residue was reconstituted in methanol. Finally, the assay procedure was followed using a portion of the final methanolic solution and reading corrected against urine blank reading.

## Result and discussion

Several analytical approaches rely on derivatizing a primary amino group by the fluorogenic reagent. Ninhydrin, NBD-Cl, NQS (1,2-naphthoquinone-4-sulfonate), acetylacetone, fluorescamine, and *o*-phthalaldehyde are the prevalent reagents probe. *o*-Phthalaldehyde is more preferred over other fluorogenic reagents due to the absence of the extraction step as in NQS and no heating step like that in ninhydrin, NBD-Cl, NQS, and acetylacetone. Moreover, the reagent has a low price compared to fluorescamine. Although the price of both NBD-Cl and *o*-phthalaldehyde are relatively similar, a very low concentration is needed in the case of *o*-phthalaldehyde (0.01%) compared to NBD-Cl (0.1%) which would efficiently reduce the cost per run. It is known that heating and extraction steps render analytical assay tedious, and lengthy.

**Fig. 1:**
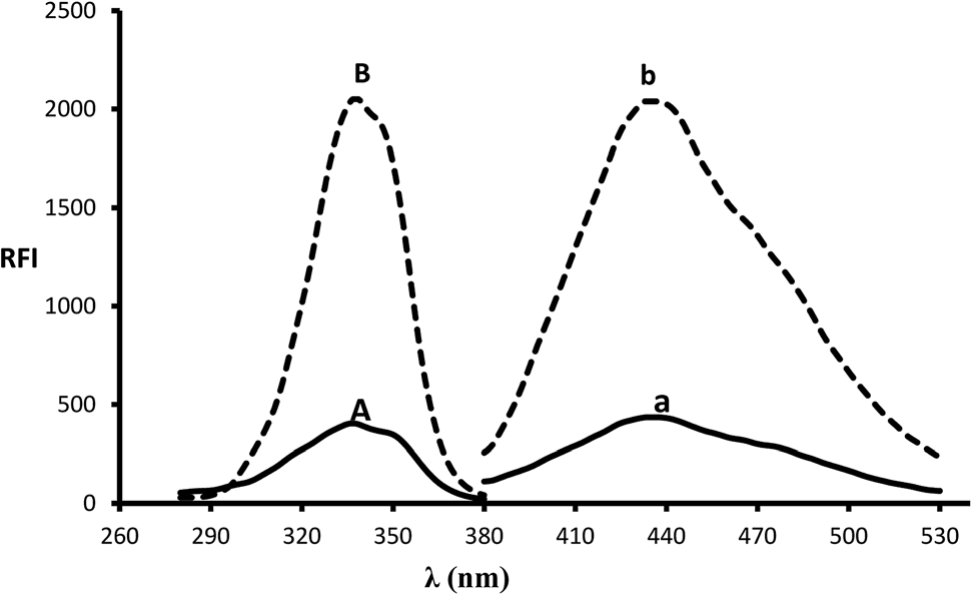
Excitation and emission spectra (B and b) of isoindole product formed from the reaction of MLC (2000 ng mL^−1^) with *o*-phthalaldehyde, in addition to blank solution (A and a).

**Scheme 1:**
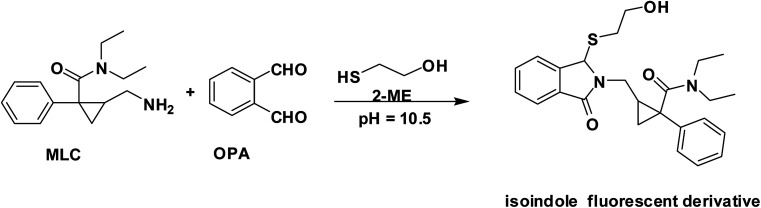
The pathway for isoindole derivative formation between MLC and *o*-phthalaldehyde/2-mercaptoethanol reagent.

In the presence of a thiol-containing compound as 2-mercaptoethanol, the amino moiety of MLC condenses with *o*-phthalaldehyde to generate isoindole fluorescent derivative. The reaction proceeded in basic pH and 2-mercaptoethanol is essential for isoindole product stability.^20^ The MLC derivative was monitored at (*λ*_ex_ 338.5 nm, *λ*_em_ 433.5 nm) (Fig. 1) and (Scheme 1) elucidate isoindole formation mechanism. Successful application of the approach on MLC containing dosage forms and biological fluids (plasma/urine) was established after complete validation of the method.

### Reaction variables optimization

The isoindole product was inspected at a variety of wavelengths to choose the accurate wavelength (excitation, emission). Furthermore, variables that affect stability and product formation were precisely studied and optimized.

### pH and borate buffer volume

**Fig. 2:**
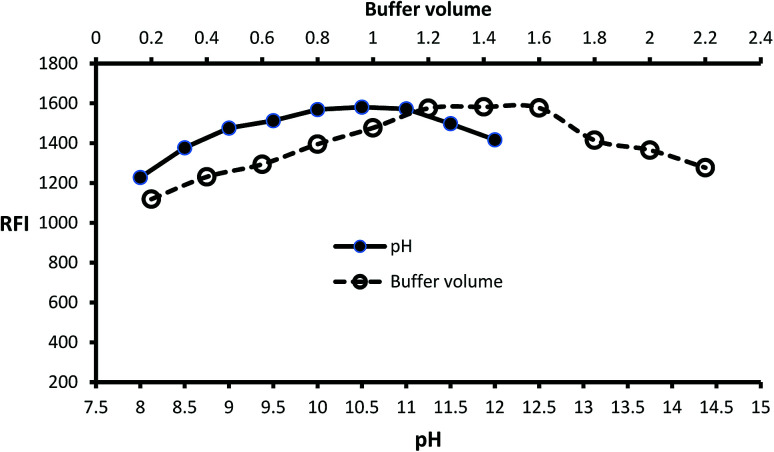
Effect of pH and buffer volume on the fluorescence strength of the isoindole product formed from MLC (2000 ng mL^−1^) with *o*-phthalaldehyde.

Inspection of the fluorescence strength of isoindole product at a variety of pH solution (8–12) was carried out by using borate buffer. Isoindole formation proceeded at alkaline pH and maximum strength attained at pH = 10.5 (Fig. 2). Furthermore, the borate buffer volume was studied by performing several experiments by different volumes (0.2–2.2 mL). The data of analysis represented in (Fig. 2) reveal that 1.4 mL is optimum.

### Reagent volume (*o*-phthalaldehyde/2-mercaptoethanol)

**Fig. 3:**
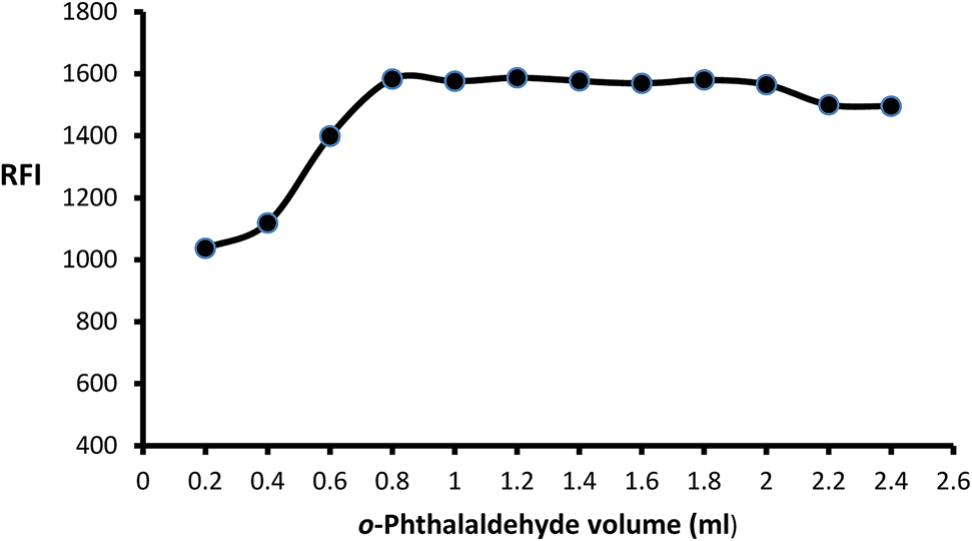
Effect of different volumes of *o*-phthalaldehyde (7.46 × 10^−4^ M) on the fluorescence intensity of isoindole product using (2000 ng mL^−1^) of MLC.

**Fig. 4:**
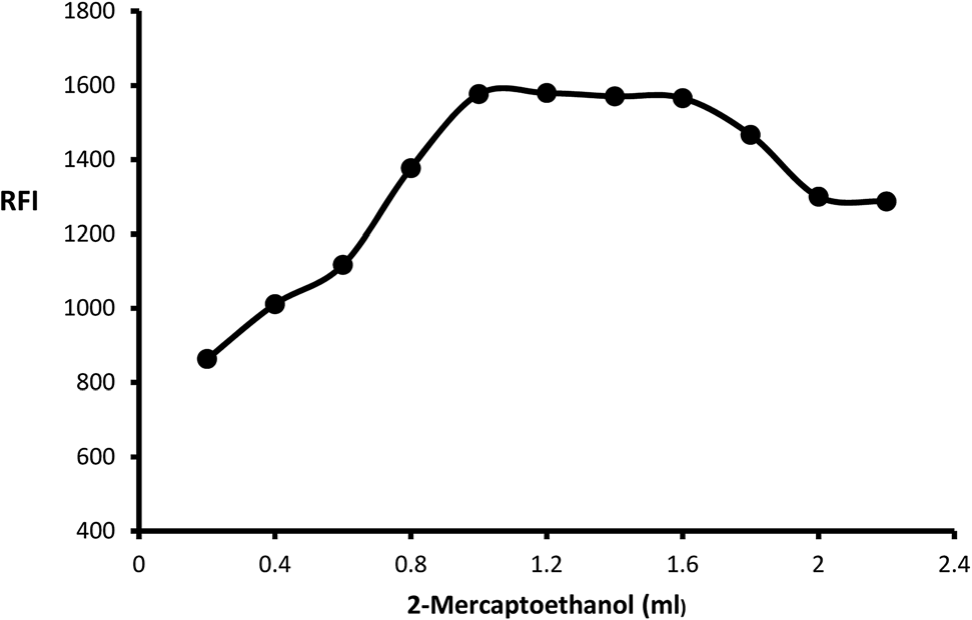
Effect of different volumes of 2-mercaptoethanol (0.01 v/v) on the fluorescence intensity of isoindole product using (2000 ng mL^−1^) of MLC.

The impact of *o*-phthalaldehyde (7.46 × 10^−4^ M) on the derivative's fluorescence was evaluated by using different volumes (0.2–2.4 mL). It was noted that FI gradually increased with increasing volume to give constant fluorescence (0.8–2 mL) followed by a slight gradual decrease (Fig. 3). To stabilize isoindole product, 2-mercaptoethanol addition is an important step, so the fluorescence strength values were recorded after adding different volumes of 2-mercaptoethanol (0.01 v/v). The high product fluorescence was recorded by adding 1.2 ± 0.2 (Fig. 4).

### Time and diluting solvents effect

**Fig. 5:**
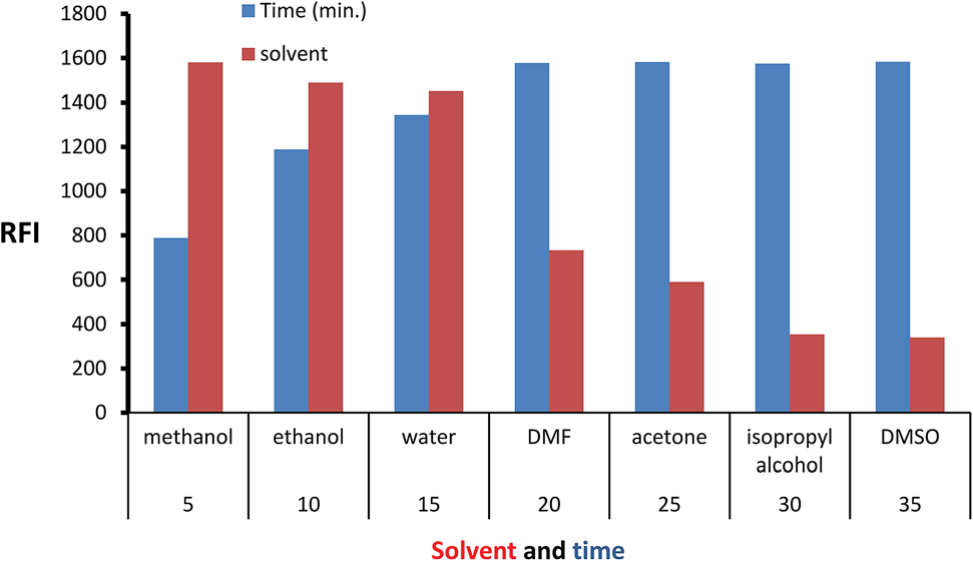
Effect of time and diluting solvents on the fluorescence intensity of isoindole product using (2000 ng mL^−1^) of MLC.

Isoindole derivative formed by the reaction of MLC with *o*-phthalaldehyde was diluted by a variety of solvent to select the appropriate one. The inspected solvents were isopropyl alcohol, water, DMSO, methanol, acetone, dimethylformamide, and ethanol (Fig. 5) exhibit high fluorescence value with methanol. The isoindole derivative was allowed to be formed at various time intervals (5–35) minutes. The values of (Fig. 5) show that 25 minutes is adequate time for product formation (isoindole derivative) with maximum FI. Finally, the effect of temperature was tested at different values and the ambient temperature was suitable for isoindole formation.

### Work validation

The procedure of the assay was strictly validated by following directives of ICH guidelines.^21^ The inspected parameters were linearity range, sensitivity (LOQ, LOD), precision, accuracy, and robustness.

### Linearity range and sensitivity

Construction of the calibration graph was established at seven standard MLC concentrations. Each MLC concentration was analyzed 3 times by the approach procedure. The fluorescence intensities values were plotted against MLC concentration to derive regression equation. The value of correlation coefficient (0.9998) indicates excellent linearity between response and MLC concentration over the range of (200–4000 ng mL^−1^). The statistical data and regression equation was found in (Table 1). The sensitivity of the proposed approach was assessed by LOQ and LOD which is estimated by using equations of ICH guidelines. The equations state that LOQ = 10 *σ*/*b* and LOD = 3.3 *σ*/*b*. It is known that (*σ*) is the intercept (SD) standard deviation while (*b*) is calibration slope. The calculated values were 150.4 and 49.6 ng mL^−1^ for LOQ and LOD respectively.

**Table 1:** Regression parameters for the proposed spectrofluorimetric method for the assay of MLC

Parameter	Spectrofluorimetric method
Linear range (ng mL^−1^)	200–4000
Slope	0.7272
SD of slope (*S*_b_)	0.005
Intercept	133.26
SD of intercept (*S*_a_)	10.94
Correlation coefficient (*r*)	0.9998
Determination coefficients (*r*^2^)	0.9997
Number of determinations	7
Limit of quantitation (ng mL^−1^)	150.4
Limit of detection (ng mL^−1^)	49.6

### Accuracy

Analysis of five MLC concentrations within the calibration curve was performed by the proposed approach. Each MLC concentration was analyzed 3 times and the results were estimated as percentage recovery and the estimated standard deviation. The data in (Table 2) exhibit excellent harmony between the estimated and actual values which confirm the accuracy of the work procedure.

**Table 2:** Assessment of the accuracy of the validated method at five concentration levels within the linear range

Taken conc. (ng mL^−1^)	Found conc. (ng mL^−1^)	Recovery^a^ ± SD	% Error
400	393.39	98.35 ± 1.39	−1.65
1200	1214.35	101.20 ± 1.74	1.20
1800	1789.61	99.42 ± 0.35	−0.58
2200	2214.07	100.64 ± 0.67	0.64
3200	3164.29	98.88 ± 0.93	−1.12

a Mean of three determinations and SD is the standard deviation.

### Precision

Intra-day precision which represents repeatability and inter-day precision were checked at five MLC concentrations within linearity. While intra-day precision was performed on the same day (triplicate measurement for each concentration), inter-day precision was verified on three consecutive days. After data processing, the calculated RSD values were lower than 2 (Table 3) which in agreement with ICH guidance and prove the procedure precision.

**Table 3:** Intra-day and inter-day precision of the proposed spectrofluorimetric method

Conc. level (ng mL^−1^)	Intraday precision		Interday precision	
	Recovery^a^ ± SD	RSD	Recovery^a^ ± SD	RSD
400	100.64 ± 1.82	1.81	98.46 ± 1.21	1.23
1000	98.33 ± 1.73	1.76	98.93 ± 1.20	1.21
2000	100.37 ± 1.73	1.72	101.44 ± 1.59	1.57
3000	99.38 ± 0.55	0.56	100.37 ± 0.65	0.64
3500	100.98 ± 1.77	1.75	101.24 ± 0.85	0.84

a Mean of three determinations and SD and RSD are the standard deviation and relative standard deviation respectively.

### Robustness

Approach robustness was studied by an intended small variation of the assay parameters. The variables varied were pH, buffer volume, *o*-phthalaldehyde, and 2-mercaptoethanol. The results presented in (Table 4) confirm that a small variation of the reaction conditions has no significant change in the results which indicates the robustness of the method.

**Table 4:** Robustness study of the proposed method for determination of MLC in pure form

Method parameters	Value	% Recovery ± SD^a^
pH	10.7	98.25 ± 1.44
	10.3	101.13 ± 0.46
Buffer volume (mL)	1.3	101.13 ± 1.55
	1.5	100.39 ± 1.28
*o*-Phthalaldehyde volume (mL)	0.9	98.96 ± 1.57
	1.1	100.76 ± 0.55
2-Mercaptoethanol (mL)	1.1	99.23 ± 0.56
	1.3	98.86 ± 1.49

a Mean of three determinations and SD is the standard deviation.

## Application

### Pharmaceutical tablets application

In the Egyptian market, MLC was formulated in many tablets formulation, among them Milnavella or Averomilan®. The proposed method was applied for the determination of MLC in the cited products by following the general assay procedure. A comparison of the obtained results with the reported fluorimetric method^9^ was performed by calculating Student's *t*-test and *F*-test to ensure accuracy and precision. At the confidence limit 95%, the calculated values were lower than the tabulated values (Table 5). This an evidence of the suitability of the approach in dosage form analysis.

**Table 5:** Comparison between the proposed and reported method^12^ for the determination of MLC in tablets^a^

Dosage form	Proposed method	Reported method^12^	*t*-value^a^	*F*-value^a^
	Recovery^a^ ± SD	Recovery^a^ ± SD		
Averomilan®	98.88 ± 1.03	99.34 ± 0.92	0.74	1.27
Milnavella	101.15 ± 1.53	100.62 ± 0.88	0.67	3.04

a Average of 5 determinations, a; the tabulated *t*- and *F*-values at the 95% confidence limit are 2.78 and 6.39, respectively.

### Approach application in spiked urine and plasma

After 2 h of MLC (50 mg) tablet administration, the maximum plasma concentration (135 ng mL^−1^) is reached. Also, about 60% of MLC excreted in the urine without changing.^9^ Approach application for MLC analysis in spiked (plasma/urine) was established and validated without any notable interference. The data in (Table 6) represent mean recovery of four spiked concentration was estimated from the corresponding regression equation shows the suitability of the approach as a preliminary test for MLC assay in spiked urine and plasma.

**Table 6:** Analysis of milnacipran hydrochloride in spiked human urine and plasma using isoindole formation reaction

Conc. (ng mL^−1^)	Spiked plasma		Spiked urine	
	Recovery^a^ ± SD	% Error	Recovery^a^ ± SD	% Error
250	101.65 ± 1.79	1.65	101.58 ± 1.43	1.58
1000	98.17 ± 1.57	−1.83	98.26 ± 1.83	−1.74
1500	98.52 ± 0.71	−1.48	101.97 ± 1.04	1.97
2000	101.32 ± 1.92	1.32	98.07 ± 1.76	−1.93

a Mean of three determinations and SD is the standard deviation.

## Conclusion

The emphasis in this work was directed in the development of a new, feasible, and sensitive fluorimetric method. The method offers many benefits like simplicity as it implemented in a one-pot without heating or extraction, low reagent price, and sensitivity. Furthermore, the measurements were monitored in a spectrofluorometer that is more preferable than other analytical instrumentation. The priority of spectrofluorometer is related to its low price, availability in most laboratories, measurement selectivity, and not require a high expert in usage or tedious sample preparation. The concept of the analytical approach relies on the condensation of MLC amino moiety with *o*-phthalaldehyde to generate isoindole fluorescent derivative in the presence of 2-mercaptoethanol. The isoindole product was monitored at (*λ*_ex_ 338.5 nm, *λ*_em_ 433.5 nm) and condensation variables were strictly optimized. Finally, the approach applied for MLC analysis in bulk powder and dosage form and involved in urine and spiked plasma analysis with excellent percentage recovery.

## Conflicts of interest

The authors declare that there is no conflict of interest.

## Supplementary Material
